# Biocompatible and Flexible Cellulose Film for the Reversible Colourimetric Monitoring of pH and Mg (II)

**DOI:** 10.3390/s26030880

**Published:** 2026-01-29

**Authors:** Iva Karneluti, Deepak Joshy, Gerhard J. Mohr, Cindy Schaude, Matthew D. Steinberg, Ivana Murković Steinberg

**Affiliations:** 1 University of Zagreb Faculty of Chemical Engineering and Technology, Marulićev Trg 19, 10000 Zagreb, Croatia; 2 Joanneum Research Forschungsgesellschaft mbH—Materials, Franz-Pichler-Straße 30, A-8160 Weiz, Austria; 3 GoSense Wireless Ltd., Cambridge CB23 6FN, UK; matthew.steinberg@gosense-wireless.com

**Keywords:** colourimetric sensors, magnesium ions, pH sensing, cellulose based sensors, metal ion detection, wearable sensors

## Abstract

**Highlights:**

**What are the main findings?**

- Fast, reversible colourimetric sensing of pH and Mg^2+^ with high selectivity over Ca^2+^ and common physiological ions.
- Transparent, flexible, and biocompatible cellulose-based thin film material suitable for continuous real-time and wearable optical sensing.

**What are the implications of the main findings?**

- Covalent immobilisation of Hyphan I chromophore on cellulose via vinylsulfonyl chemistry.
- Stable, non-leaching sensor platform enabling scalable and cost-effective fabrication.

**Abstract:**

Novel colourimetric sensors are readily devised by combining multifunctional (nano)materials with miniature optoelectronic components. The demand to detect and monitor metal ions has resulted in the invention of new colourimetric sensing schemes, especially for use at the Point-of-Need (PoN). Nonetheless, the design of fully reversible optical materials for continuous real-time ion monitoring remains a bottleneck in the practical realisation of sensors. Magnesium ion is vital to physiological and environmental processes, but monitoring can be challenging, particularly in the presence of Ca^2+^ as a cross-sensitive interferent in real samples. In this work, a chromophore molecule Hyphan I (1-(2-hydroxy-5-ß-hydroxyethylsulfonyl-phenyl-azo)-2-naphthol) has been grafted onto a cellulose matrix with a simple one-pot vinylsulfonyl process, to form a transparent, biocompatible and highly flexible thin-film colourimetric magnesium ion sensing material (Cellulose Film with Hyphan-CFH). The CFH film has a pH response time of <60 s over the pH range 4 to 9, with a p*K*_a1_ = 5.8. The LOD and LOQ for Mg^2+^ at pH 8 are 0.089 mM and 0.318 mM, respectively, with an RSD = 0.93%. The CFH film exhibits negligible interference from alkaline and alkaline earth metals, but irreversibly binds certain transition metals (Fe^3+^, Cu^2+^ and Zn^2+^). The CFH material has a fast and fully reversible colourimetric response to pH and Mg^2+^ over physiologically relevant ranges without interference by Ca^2+^, demonstrating good potential for integration into microfluidic systems and wearable sensors for biofluid monitoring.

## 1. Introduction

The availability of new multifunctional (nano)materials and optoelectronic components has resulted in much research on novel colourimetric sensors for the detection and monitoring of analytes, especially for use at the Point-of-Need (PoN) [1,2,3,4,5,6]. The applications of colourimetric sensors range from biomedicine, Point-of-Care (PoC) diagnostics, wearable devices, to food safety, agricultural, and environmental monitoring; there is a broad demand for functional sensors from these markets. Despite recent advances in rapid manufacturing technologies and ease of miniaturisation to micron scale features, the functional design of colourimetric materials that are both reversible and selective remains a challenge to the realisation of real-time optical chemical sensors as integrated analytical devices. Here, we address some of the functional challenges with the introduction of a biocompatible and flexible colourimetric thin-film sensor with fully reversible response to magnesium ions and pH.

Metal ions play a vital role in the regulation of biological and environmental processes [7,8], so monitoring of their presence and concentration is important across many application areas. Magnesium ion is vital to physiological processes and organisms [9], and for environmental [10] and agricultural balance in natural systems [11]. Besides the use of established ion-selective electrodes for the detection and quantification of Mg^2+^ [12], various optical sensors have been reported [13]. These include colourimetric [14,15], fluorescent [16,17,18], and surface plasmon-based devices [19]. The advantages of the simpler colourimetric sensors include a visible colour change that may be interpreted by the naked eye, operation with low-cost and portable instrumentation such as colour metres and photometers, may be mass produced on sustainable/green substrate materials such as paper and textiles, and may be used with commonly available mobile phone cameras [20,21,22,23,24]. Metal ion sensors can suffer from irreversible binding to the target ion and cross-sensitivity to interferent ions present in the sample due to strong binding constants. Reversibility may nonetheless be achieved with the right combination of substrate, indicator, analyte, and immobilisation technique [25]. Choice of substrate material is therefore important and influences the ultimate reversibility of the sensor, its biocompatibility and wearability. Lastly, it is known that Mg^2+^ ion sensors often suffer from cross-sensitivity and interference to Ca^2+^ that is present in body fluids and environmental samples which is problematic for calibration [13].

Colourimetric sensors for metal ions may utilise established reversible ionophore systems like ion-selective optodes [26], gold and silver nanoparticle platforms [27], and organic chromophores [28]. Organic chromophores for pH and metal ion-sensing are typically Brønsted acidic/basic dyes or Lewis acid/base dyes, respectively, that change their absorption properties upon (de)protonation or complexation with metal ions. Ionochromic dyes are strong coordination ligands for colourimetric complexation of metal ions, and their immobilisation into polymer substrates is crucial in the design of stable sensing materials. Of the available immobilisation methods, covalent immobilisation of indicator dye directly to the solid substrate provides the greatest stability and durability and minimises leaching of the dye [29,30].

One of the most biocompatible and biodegradable sensor substrate materials is cellulose [31,32], although many biopolymers and their hydrogels are known to be suitable [33,34,35,36]. But the natural abundance, surface hydrophilicity, enhanced analyte diffusion, and high density of surface hydroxyls make cellulose a primary choice of substrate material [37,38]. Cellulose can be used in a variety of forms, including paper, film and nanofibers, moreover regenerated cellulose-based substrates like cellophane have good transparency, mechanical strength, and are likely compatible with microfluidics—all of which are prerequisites for next-generation wearable sensors and continuous real-time monitoring [39].

Established metal-ion indicators containing an *o,o*’ dihydroxy azobenzene complexation moiety, such as Eriochrome Black T (EBT) and Eriochrome Blue Black R (EBB), have been reported for the determination of transition metal ions, for Mg^2+^ and Ca^2+^ and for water hardness testing [40,41,42,43]. The structurally similar, but less well explored chelator, Hyphan I [44] has previously been used for the extraction of transition metal ions from complex mixtures [45]. Here, Hyphan I, 1-(2-hydroxy-5-ß-hydroxyethylsulfonyl-phenyl-azo)-2-naphthol, is used as a precursor for covalent immobilisation on cellulose. We anticipated that the combination of Hyphan I, a multifunctional chelating molecule, with a cellulose substrate would result in a new sensing material with enhanced optical properties and appropriate p*K*_a_ and binding constants for reversible response. Hyphan I was therefore grafted onto cellulose in a simple one-pot vinylsulfonyl process to form a transparent, biocompatible, and highly flexible thin-film colourimetric sensing material (Cellulose Film with Hyphan-CFH). The characterisation of CFH as an ion sensor shows a fast and fully reversible response to pH and Mg^2+^ in physiologically relevant ranges without cross-sensitivity to Ca^2+^. This work demonstrates the future potential of CFH-based sensors to function as wearable and in-line microfluidic analytical devices for biofluid monitoring.

## 2. Materials and Methods

### 2.1. Chemicals

Chemicals for the synthesis of the dye were of reagent grade while chemicals for the immobilisation of the dye to cellulose (concentrated sulphuric acid, sodium hydroxide, sodium carbonate), buffers and chloride salts of alkali and alkaline earth metal ions for spectral evaluation (tris(hydroxymethyl)aminomethane (Tris), sodium dihydrogen phosphate, sodium acetate, sodium sulphate, boric acid, magnesium chloride hexahydrate, calcium chloride dihydrate, sodium chloride and potassium chloride) were all of analytical reagent grade. 2-Amino-4-(2-hydroxyethylsulfonyl)-phenol was obtained from Merck. The regenerated cellulose layers with a thickness of 35 µm were from Innovia (NatureFlex^TM^ 35 NP), Futamura Chemical Co. Ltd., Nakamura, Japan.

### 2.2. Synthesis of the Chromoionophore Hyphan I

The synthesis was performed according to a procedure described by Burba [44]. Here, 1.47 g (6.8 mmol) of 2-amino-4-(2-hydroxyethylsulfonyl)-phenol was suspended in 2.2 mL (13.2 mmol) of 6 N hydrochloric acid and an additional 1.4 mL of distilled water and cooled to below 5 °C. To this, a solution of 0.28 g (4.1 mmol) of sodium nitrite in 2 mL of distilled water was added, and the resulting orange-brown suspension was stirred for 20 min at 5 °C and filtered. This filtrated diazotisation solution was slowly added to an ice-cooled solution of 0.58 g (4.0 mmol) of 2-naphthol previously dissolved in 2 mL of ethanol and added to 0.2 g (5.0 mmol) of sodium hydroxide and 1.0 g (9.4 mmol) of sodium carbonate in 20 mL of distilled water. The resulting mixture was stirred for 3 h. Then, it was acidified with 5 mL of 6 N hydrochloric acid to precipitate the red-brown dye. Column chromatography using dichloromethane/acetone (2:1) as the eluent gave red crystals. ^1^H-NMR (DMSO): d (ppm) 16.24 (s, 1H, -OH), 11.85 (s, 1 H, -OH), 8.43 (d, 1 H, =CH-), 8.32 (s, 1 H, =CH-), 7.93 (d, 1 H, =CH-), 7.67 (m, 3 H, =CH-), 7.47 (d, 1 H, =CH-), 7.20 (d, 1 H, =CH-), 6.78 (d, 1 H, =CH-), 4.93 (s, 1 H, -OH), 3.73 (t, 2 H, -CH_2_-), 3.52 (t, 2 H, -CH_2_-) (Supplementary Materials Figure S1). Mass spectral analysis: 373.0 Da [MH^+^] (Supplementary Materials Figure S2). Yield: 20%.

### 2.3. Fabrication of Sensor Layers

Hyphan I indicator molecules were immobilised on transparent cellulose film (CFH) following the common procedure used for transparent cellulose, textiles, and wipes [46]. In a typical immobilisation procedure, 50 mg of the dye was treated with 0.5 mL of concentrated sulfuric acid for 30 min at room temperature. This converts the hydroxyethylsulfonyl group of the indicator dye into the corresponding sulfonate. The sulfonated mixture was then poured into 400 mL of distilled water, and 1 mL of 32% sodium hydroxide solution was added to it for neutralisation. After placing the cellulose film for 5 min in this solution, 12.5 g of sodium carbonate in 100 mL of water was added to it, followed by the addition of 2.5 mL of 32% sodium hydroxide solution after 5 min. The sulfonated dye was converted into the chemically reactive vinylsulfonyl derivative in the prevailing basic condition, and in turn, vinylsulfonyl groups underwent Michael addition with the hydroxyl groups of the cellulose film. After 30 min, the indicator immobilised cellulose layers were removed from the dyeing bath and washed with distilled water. 

### 2.4. Measurements

Mass spectral analysis of the synthesised Hyphan I indicator was carried out using an Agilent 6550 Series Accurate-Mass-Quadrupole Time-of-flight system, Santa Clara, CA, USA, and ^1^H-NMR (DMSO) spectra were obtained using a Bruker Avance III HD 400 MHz, Rheinstetten, Germany. For the pH-dependent sensing studies, Britton–Robinson and Tris buffer solutions were used. The wide pH range Britton–Robinson buffer was prepared using 0.04 M sodium acetate, 0.04 M boric acid, 0.04 M sodium dihydrogen phosphate, and 0.1 M sodium sulphate. For the interference-specific studies at pH 7.4 and 8.0, 50 mM Tris buffer was employed. 1.0 M aqueous sodium hydroxide and 1.0 M aqueous hydrochloric acid were used for pH adjustments of the buffer solutions. pH measurements were carried out using a WTW pH electrode SenTix 62, Xylem Analytics, Weilheim, Germany. The optical responses of the Hyphan I immobilised cellulose (CFH) film corresponding to various pH and metal ion concentrations were collected using a Shimadzu UV-1280 UV-visible spectrometer, Kyoto, Japan, in the absorbance mode. For this, the CFH film was cut according to the cuvette dimensions and placed against the cuvette wall, followed by the addition of different pH buffer solutions and metal ion concentrations into the cuvette. The sensing responses were then gathered by collecting the absorbance spectra.

## 3. Results and Discussion

### 3.1. Choice of Sensing Material

**Figure 1:**
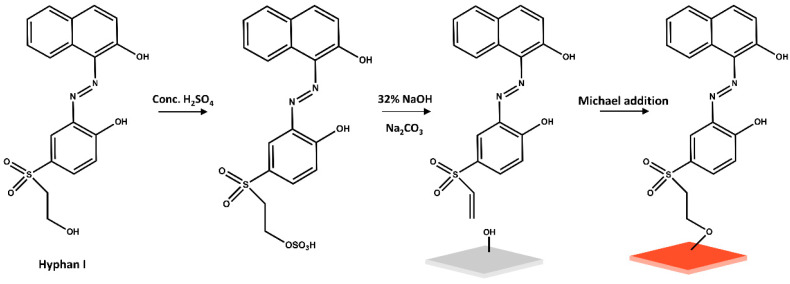
Hyphan I immobilisation on a pristine cellulose film (grey) to obtain the CFH sensitive material (red).

The colourimetric sensing material designed for this study was fabricated by covalent immobilisation of the azo indicator dye Hyphan I, 1-(2-hydroxy-5-ß-hydroxyethylsulfonyl-phenyl-azo)-2-naphthol, onto transparent cellulose films, Figure 1.

The dye contains naphtholic and phenolic hydroxyl groups, providing pH and metal ion complexation sites, while the hydroxyethylsulfonyl group at the end of the molecule can be used for grafting onto a cellulose film. Hyphan I is the same class of naphthol-type pH indicator dye together with Nitrazine Yellow and Naphthol Orange. It can serve as a pH indicator due to its ability to change colour with varying pH conditions. The dye molecule contains an *o,o*’-dihydroxy azobenzene moiety, similar to Eriochrome Black T (EBT), Eriochrome Blue Black R (EBB) and Calmagite indicators, that are known for complexation of heavy metal and alkaline earth metal ions and which are used in various applications, including the removal of heavy metal ions from drinking water [40,42,44].

The covalent immobilisation of the dye is based on a simple one-pot vinylsulfonyl chemistry, schematically shown in Figure 1 [46,47,48]. Typically, the molecule is first converted to a sulfonate in acidic conditions, followed by conversion to a chemically reactive vinylsulfonyl derivative under basic conditions. The vinylsulfonyl groups react with hydroxyl groups of the cellulose via Michael addition, providing a covalent attachment of the molecule onto the cellulosic material.

The resulting sensing material, CFH, is a 35 μm thick, transparent, biocompatible, and highly flexible cellulose film covalently functionalised with Hyphan I colourimetric indicator.

It is known that in most cases, indicators retain their complexation properties upon immobilisation; however, with altered selectivity, sensitivity, reversibility, and response times, characteristics are also strongly related to the physical and chemical properties of the substrate [25,31]. Cellulose is a natural biopolymer and is non-cytotoxic when functionalised with covalently bound azo-indicator [49]. This makes it compatible for use in wearable and epidermal monitoring applications [50,51,52].

The following steps of this study included characterisation of CFH as a potential pH and metal ion sensing material.

### 3.2. pH Sensitivity of the CFH Sensing Layer

**Figure 2:**
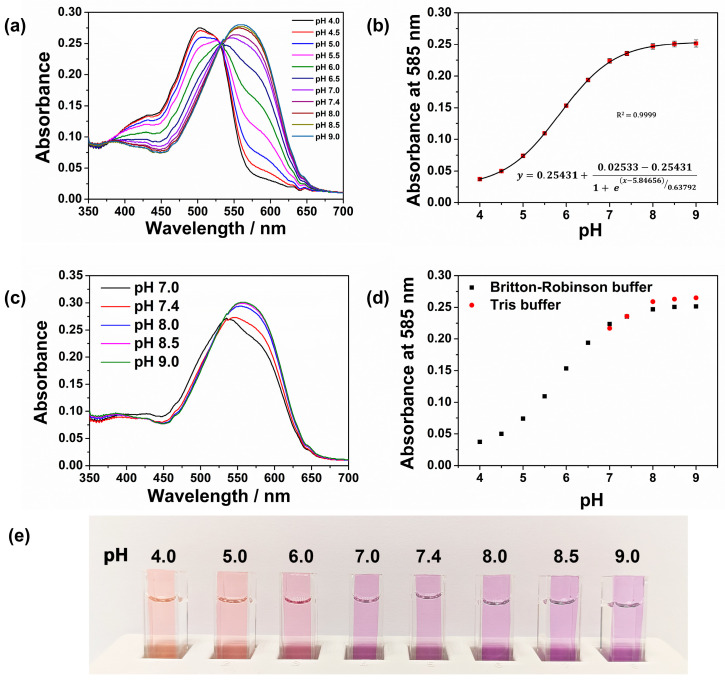
(**a**) UV-Vis spectra of the CFH film in Britton–Robinson buffer in the range of pH 4.0–9.0 (**b**) calibration curve fitted with Boltzmann model for the pH response of the film in the range of pH 4.0–9.0; *n* = 6 (**c**) UV-Vis spectra of the CFH film in Tris buffer in the range of pH 7.0–9.0 (**d**) comparison of the pH sensing responses in Britton–Robinson and Tris buffers (**e**) visible colour change in the CFH film in different pH buffer solutions.

The spectral pH sensing performance of the cellulose film CFH was tested in pH buffers over the pH range 4.0–9.0, Figure 2.

**Figure 3:**
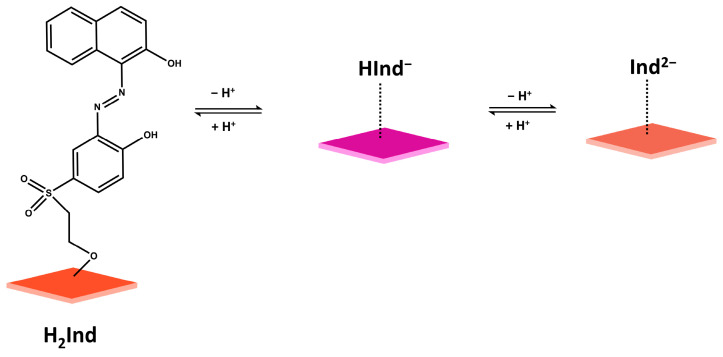
Proposed pH-dependent equilibria of the cellulose film immobilised Hyphan I (CFH) sensor.

The UV-visible absorption spectra show a bathochromic shift upon deprotonation of the hydroxyl group in Hyphan I, from 502 nm to 560 nm with a clear isosbestic point at 532 nm. This is manifested as a visible colour change in the sensor layer from orange-red to purple, as proposed by the equilibrium 1, Figure 3. It is known that both phenolic groups in similar molecules, EBT and Calmagite, are protonated at pH < 6 [41]. Given their similar structure, deprotonation of the Hyphan I molecule most likely follows the same deprotonation scheme, as shown in Figure 3. The absorption spectra of CFH at pH 8.0–12.5 are given in Supplementary Materials Figure S3. The shift in absorption spectra in that range corresponds to deprotonation of the second -OH group, with an apparent p*K*_a2_ around 12.

However, it is known that both the acid-base and the tautomeric azo-hydrazone equilibria occur within the *o,o*’-dihydroxy azobenzene moieties in aqueous solutions, as analysed in the case of Calmagite [53]. Such complex protropic equilibria may additionally affect the indicator spectral properties when in immobilised form.

Deprotonation of the first hydroxyl group causes a shift in the absorption peak to a longer wavelength (from 502 nm to 560 nm), which is expected and can be explained by the enhanced electron donor strength of the anionic -O^−^ phenolate group relative to the phenolic hydroxyl group -OH [54]. The film responds to pH changes reversibly, with a response time of less than 1 min. The corresponding pH equilibrium constant, p*K*_a1_ = 5.84, is calculated from the calibration curve fitted with the Boltzmann model, Equation (1), for six repetitive cycles of pH measurements using the same film, Figure 2b and Supplementary Materials Table S1. The standard deviations and relative standard deviations corresponding to each pH were found to be less than 0.0058 and 4.0%, respectively. The regression coefficient, *R*^2^ = 0.9999, confirmed good fit of the experimental data to the theoretical Boltzmann model.

(1) y=A2+A1−A21+e(x−x0)dx

The spectral response of the CFH film in the range pH 7.0–9.0 was also tested in Tris buffer, Figure 2c. Tris buffer is used in biological analysis due to its minimal ion content. The corresponding pH titration plots in Britton–Robinson and Tris buffers are shown in Figure 2d. The optical responses observed in the two buffers are in good agreement, with the small differences ascribed to the different ionic compositions. It is interesting to note that at pH 7.4, which corresponds to physiological pH, the optical response of CFH in each buffer coincides.

### 3.3. Response to Metal Ions

**Figure 4:**
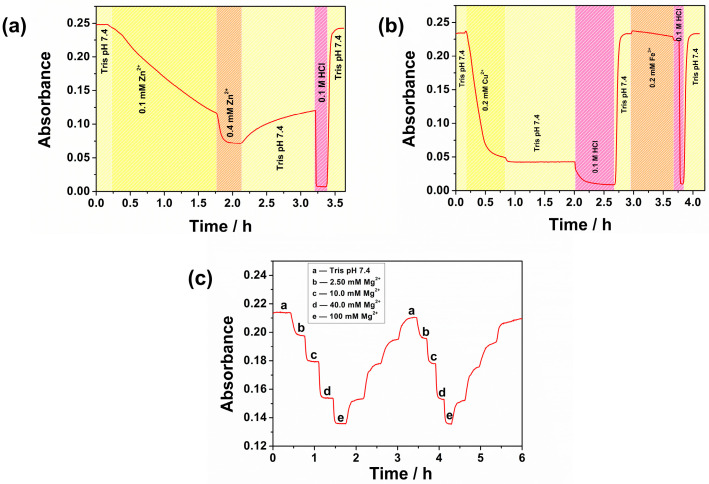
Dynamic flow-through cell reversibility studies of CFH film at varying concentrations of (**a**) Zn^2+^ (0.10 and 0.40 mM), (**b**) Cu^2+^, and Fe^3+^ (0.20 mM each), (**c**) Mg^2+^ (2.50, 10.0, 40.0 and 100 mM) in pH 7.4 Tris buffer.

Eriochrome Blue Black R is a structurally equivalent ligand to Hyphan I and forms complexes with different transition metals, amongst which Zn^2+^, Cu^2+^, and Fe^3+^ show the highest formation constants [43]. The response of CFH towards these ions was investigated in a flow-through cell at pH 7.4, Figure 4a,b.

Figure 4a shows the change in absorbance at 585 nm with increasing Zn^2+^ concentration from 0.1 to 0.4 mM. The response is not reversible, and after 2 h, the initial signal of the CFH could not be recovered with buffer at pH 7.4. However, complete recovery of the initial baseline signal was achieved with 0.1 M HCl. A similar trend in reversibility was observed for Cu^2+^ and Fe^3+^ ions too, Figure 4b. The flow-through cell experiments confirmed the strong, irreversible binding of transition metal ions to the CFH film.

Unlike the transition metal ions, Mg^2+^ exhibited a fully reversible response in a concentration range from 2.50 mM to 100 mM over two consecutive reversible cycles lasting 6 h, Figure 4c. This unique reversible binding affinity of the CFH towards Mg^2+^ ions can be partially attributed to its smaller ionic size, higher charge density, and higher hydration energy [55,56] and resembles typical behaviour of an ionophore based sensing system. In such systems ion selectivity is usually governed by cavity size and donor atom type, including their structural factors, solvation and desolvation energies, complex stability, electron density distribution, and conformational rigidity [57]. In the case of CHF, the right combination of cellulose matrix structural features, including its polarity and porosity, with the chelator geometry and availability of oxygen coordination sites as hard bases (HSAB principle), result in an apparent complex binding constant leading to a reversible, equilibrium driven response to Mg(II). At the same time, transition metal ions like Fe^3+^, Cu^2+^, and Zn^2+^, having higher formation constants with azo dyes, can engage in stronger chelation involving both phenolate oxygen and azo nitrogen, leading to irreversible binding.

### 3.4. Response to Mg^2+^

#### 3.4.1. Effect of pH on Mg^2+^ Response

**Figure 5:**
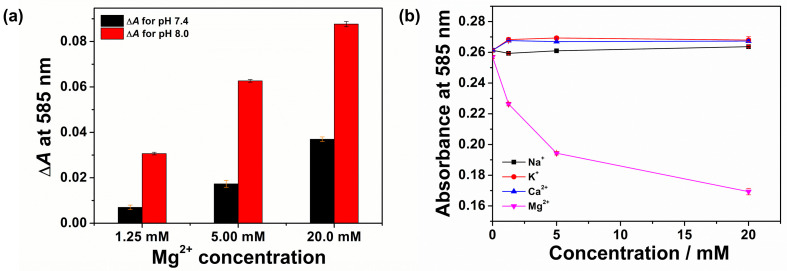
(**a**) Change in optical absorbance (Δ*A*) at 585 nm for CFH film at pH 7.4 and pH 8.0 at 1.25, 5.00, and 20.0 mM Mg^2+^ (**b**) Absorbance response of CFH film to three physiologically and environmentally relevant alkali and alkaline earth metal ions compared to Mg^2+^ at pH 8.0.

The absorbance of CFH film at Mg^2+^ concentrations of 1.25, 5.00 and 20.0 mM was studied at pH 7.4 and 8.0. The change in absorbance (Δ*A*) measured in triplicate at 585 nm is shown in Figure 5a. Δ*A* is the difference in absorbance at 585 nm between the plain buffer and the Mg^2+^ buffer solution.

Greater changes in absorbance were observed at pH 8.0 compared to pH 7.4 for all three Mg^2+^ concentrations. The increased response at pH 8.0 is the result of deprotonation of the Hyphan I phenolic -OH groups, which in turn increases the fraction of coordination sites available for Mg^2+^. Therefore, further studies were carried out at pH 8.0.

#### 3.4.2. Effect of Na^+^, K^+^, Ca^2+^ on Mg^2+^ Response

Three known interferent alkali and alkaline earth metal ions commonly present in physiological environments (Na^+^, K^+^, Ca^2+^) were tested together with Mg^2+^, in Tris buffer at pH 8.0, and the corresponding optical responses were measured in triplicate, Figure 5b.

For each alkali/alkaline earth metal ion, the optical absorbance was determined at three different concentrations: 1.25, 5.00, and 20.0 mM. The responses generated by Na^+^, K^+^ and Ca^2+^ were compared to those of Mg^2+^. At pH 8.0, the measured absorbance change for 20 mM of Mg^2+^ was found to be −34.1% of the initial blank, while Na^+^, K^+^ and Ca^2+^ showed changes of +1.40%, +2.55% and +0.75%, respectively.

The lack of a response to Ca^2+^ was unexpected, given its strong complexation with the structurally related Eriochrome indicators. The high selectivity to magnesium over calcium ion is a considerable advantage of the CFH material in comparison with similar colourimetric, indicator-based systems, especially for application in biofluids where the typical concentration of these ions is usually similar [13].

#### 3.4.3. Dynamic Range and Calibration Plots

**Figure 6:**
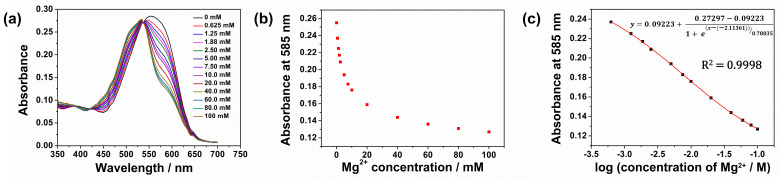
(**a**) UV-visible absorption spectra of CFH film for Mg^2+^ ion concentration 0–100 mM, (**b**) absorbance of CFH film at 585 nm for Mg^2+^ ion concentration 0–100 mM, (**c**) calibration plot of CFH film by Boltzmann curve fit.

The UV-visible absorbance spectra of CFH film in Mg^2+^ solutions (0 to 100 mM) were measured, Figure 6a. The absorbance around 585 nm decreases with increasing Mg^2+^ concentration, Figure 6b. The absorbance values at 585 nm were fitted to a Boltzmann model with a regression coefficient *R*^2^ = 0.9998, Figure 6c.

Even though the CFH exhibited a dynamic range up to 100 mM for Mg^2+^, the most sensitive and more physiologically relevant range is to 20 mM. The choice of working range depends on various factors such as required accuracy, reproducibility, reversibility, and the field of application. Based on these considerations, the CFH was evaluated from 0 to 5 mM of Mg^2+^. This has physiological relevance since Mg^2+^ in human sweat typically lies in this concentration range.

**Figure 7:**
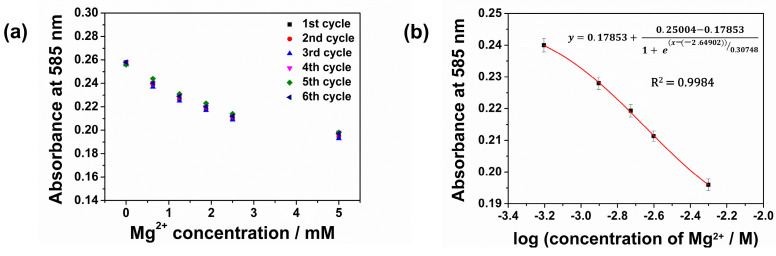
(**a**) Optical response of CFH film over six repetitive cycles in the range 0–5 mM Mg^2+^ (**b**) calibration curve fitted with the Boltzmann model.

The repeatability of the CFH is shown, Figure 7a. Here, the absorbance was measured over 6 repeated cycles (*n* = 6) in increments from 0, 0.625, 1.25, 1.875, 2.5 to 5.0 mM Mg^2+^ ion concentration (Supplementary Materials Table S2). The standard deviation and relative standard deviation corresponding to each Mg^2+^ concentration were found to be less than 0.0021 and 0.93%, respectively. Upon fitting the data to a Boltzmann model, a calibration plot was obtained with a regression coefficient *R*^2^ = 0.9984, Figure 7b. The calibration function is given by Equation (2).

(2) y=0.17853+0.25004−0.178531+e(x−(−0.264902))0.30748

#### 3.4.4. Reversibility

**Figure 8:**
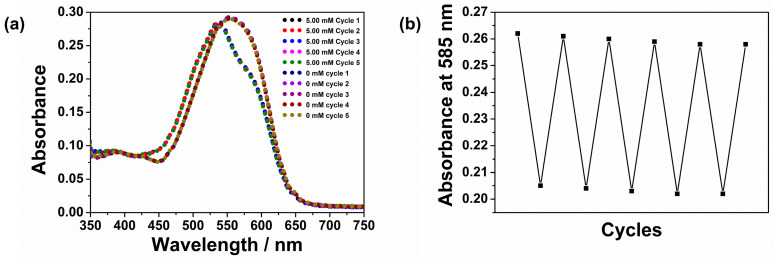
(**a**) Reversibility of the CFH film in a static cuvette test at 0 mM and 5 mM Mg^2+^ (**b**) absorbance response of the CFH film to alternating concentrations of 0 and 5 mM Mg^2+^ in Tris buffer, pH 8.0.

Reversibility of ion sensors is necessary for continuous real-time monitoring applications and is an important feature required of wearable sensors. The reversibility of the CFH to Mg^2+^ ion under dynamic flow conditions was described in Section 3.3. In addition, the reversibility of the CFH was evaluated in static cuvette tests. The CFH was alternately exposed to 0 and 5 mM Mg^2+^ solutions in Tris buffer at pH 8.0 over five repeated cycles, and the corresponding UV-visible absorption spectra measured, Figure 8a. The absorbance at 585 nm obtained over the five alternating cycles is shown, Figure 8b. The CFH was observed to be reversible in the static cuvette tests, with a relative standard deviation of less than 0.57% at 0 mM and 5 mM.

The LOD and LOQ of the CFH for Mg^2+^ at pH 8.0 are 0.089 mM and 0.318 mM, respectively, with an RSD of 0.93% (Supplementary Materials Section S3). The CFH exhibits a response time < 2 min to Mg^2+^ ions in solution and has a stable and reversible response over 6 h duration in a flow-through cell, Figure 4c. In addition, we found that CFH remains functional over several years when stored in the dark under room temperature conditions, indicating it has a good shelf-life. The performance of CFH is compared with other relevant Mg^2+^ sensors reported in the literature, Table 1. This shows how CFH combines fast response (~2 min) and reversible behaviour over the millimolar range, offering a platform for simple, reusable Mg^2+^ monitoring in aqueous samples.

#### 3.4.5. Real Sample Measurements

To evaluate the analytical performance of the CFH, three samples containing Mg^2+^ were tested. The first sample was a laboratory-prepared solution having 0.625 mM Mg^2+^ along with 5 mM of each Na^+^, K^+^ and Ca^2+^ ions. The other two samples were commercially available mineral water samples: Rommerquelle^®^ and Mg^++^ Mivela^®^. All three samples were tested for Mg^2+^ after adjusting the pH to 8.0. The absorption at 585 nm was converted to concentration using the calibration function, Table 2. The colourimetric response of the CFH in mineral water Mg^++^ Mivela^®^ is shown in Supplementary Materials Figure S4, and the Mg^2+^ concentrations corresponding to the observed optical responses were found to agree with the declared values with a maximum relative error of 5.6%. Furthermore, a spike and recovery measurement was performed in triplicate to assess method effectiveness in the presence of physiological electrolytes. A solution containing 2.5 mM of Na^+^, K^+^, Ca^2+^, and Mg^2+^ was spiked with an additional 0.646 mM Mg^2+^, yielding an average recovery of 90.55%.

When it comes to implementation of CFH system for specific applications, the composition of the sample matrix has to be considered very carefully and if the presence of transition metal ions in the sample matrix is unavoidable, some additional steps may need to be applied to remove their interference. These could involve pre-treatment of the sample to remove the effect of interfering ions [63], or adaptation of sensing material architecture using additional layers/adsorbents containing masking reagents that strongly bind those ions, e.g., chelators like 4-(2-pyridylazo)resorcinol (PAR) and 1-(2-pyridylazo)-2-naphthol (PAN) [31,64]. In addition, regular regeneration of the sensing film with acidic solutions to rinse irreversibly bound transition metal ions may be recommended.

## 4. Conclusions

In this work, a new and fully reversible optical sensor for pH and Mg^2+^ ions is demonstrated. Optical detection is based on a novel colourimetric responsive material, cellulose film with Hyphan I (CFH). To fabricate the CFH film, Hyphan I indicator is covalently immobilised on cellulose through the vinylsulfonyl group to hydroxyl groups present on the cellulose via a Michael addition. The resulting colourimetric film is transparent, thin, flexible and biocompatible with good optical properties. The CFH has a colourimetric response to pH and Mg^2+^ ions in solution, with an LOD and LOQ to Mg^2+^ of 0.089 and 0.318 mM, respectively, over a sensing range of 4.0–9.0 pH units with a response time of < 60 s. The fast reversible colourimetric response and high selectivity to Mg^2+^ compared to Ca^2+^ and other common physiological ions make the CFH suitable for Mg^2+^ sensing in biomedical applications. However, CFH also shows strong irreversible binding to Zn^2+^, Cu^2+^, and Fe^3+^ and this should be taken into account in applications where transition metal ions might be present in the sample, such as sweat. In such applications, additional steps, e.g., sample pre-treatment using non-selective masking of transition metal ions, and/or regeneration of sensing films in acidic solutions may be considered. Unlike many chelation-based sensors, where strong binding results in an irreversible response to metal ions, the CFH is fully reversible to Mg^2+^ in the physiological range up to 5 mM. This is the result of a synergistic combination of the covalently immobilised Hyphan I with the cellulose film microenvironment, where cellulose characteristics such as hydrophilicity, ion diffusion rate and surface hydroxyl density influence the optical properties and sensing performance of the indicator. The covalent immobilisation strategy ensures long-term stability of the sensor by preventing indicator leaching, and the simple vinylsulfonyl fabrication process is suitable for industrial scale-up. The CFH film demonstrates the necessary characteristics of a pH and Mg^2+^ ion-responsive optical sensor for continuous real-time monitoring and is suitable for incorporation into wearable devices.

## Figures and Tables

**Table 1:** Comparison of CFH with other Mg^2+^ sensors reported in the literature.

Sensor Type	Transduction Mechanism	Ionophore/ Chromophore Used	LOD	Working Range	Selectivity	Response Time	Reversibility	Target Sample Matrix	Ref.
ISE	Potentiometric	Esomeprazole–magnesium	4.13 × 10^−6^ mol L^−1^	1.41 × 10^−5^ mol L^−1^ to 1 × 10^−2^ mol L^−1^	Selective	8–10 s	Reversible	Drug substance	[58]
Three-dimensional printed ISE	Potentiometric	Magnesium ionophore I (C49H94N6O6)	0.039 mM	0.039–10 mM	Selective	-	Reversible	Biofluids	[59]
ISE	Potentiometric	MgI-1	-	1 × 10^−4^ to 1 × 10^−2^	Partially selective	-	Irreversible	Aqueous solutions	[60]
Optode	Fluorescence	2-(2-hydroxy-3-naphthyl)-4-methylbenzoxazole (HNBO) fluorophore	6.0 × 10^−7^ M	0–1.2 × 10^−5^ M	Selective	-	-	Aqueous solutions	[18]
Microfluidic paper-based sensor	Colourimetric	Xylidyl Blue with EGTA	0.04 mM	0 to 1 mM	Selective	10 min	Irreversible	Human serum	[61]
Wearable microfluidic colourimetric detection device	Colourimetric	Chrome Black T	0.4 mM	0.5–8 mM	Selective in the presence of Na^+^, K^+^ and Ca^2+^	10 min	Irreversible	Sweat	[62]
CFH (Present work)	Colourimetric	Hyphan I	0.089 mM	0.625–5 mM	Selective in the presence of Na^+^, K^+^ and Ca^2+^ Interference by Fe^3+^, Cu^2+^, Zn^2+^	2 min	Reversible	Aqueous solution	

**Table 2:** Comparison of declared and measured Mg^2+^ concentrations in bottled mineral water samples using the CFH.

Sample	*c*(Mg^2+^)/mM Declared	*c*(Mg^2+^)/mM Measured with CFH	Relative Error/%
Laboratory-prepared sample	0.625	0.622	0.5%
Rommerquelle^®^	2.50	2.36	5.6%
Mg^++^ Mivela^®^	4.70	4.50	4.2%

## Data Availability

The original contributions presented in this study are included in the article. Further inquiries can be directed to the corresponding authors.

## Supplementary Materials

The following supporting information can be downloaded at: https://www.mdpi.com/article/10.3390/s26030880/s1, Figure S1: ^1^ H-NMR spectrum of Hyphan I. Figure S2: Mass spectrum of Hyphan I. Figure S3: Absorption spectra of Hyphan I corresponding to its various protonated and deprotonated forms. Figure S4: Colourimetric response of the CFH towards commercially available mineral water Mg^++^ Mivela^®^. Section S1. pH response. Table S1: CFH absorbance vs. pH, 6 repetitions (R1–R6). Section S2. Mg^2+^ response. Table S2: CFH absorbance vs. Mg^2+^ in solution pH 8.0, 6 repetitions (R1–R6). Section S3: Calculation of LOD and LOQ.
