# Supplementary material for: Biocompatible and Flexible Cellulose Film for the Reversible Colourimetric Monitoring of pH and Mg (II)
Source: Sensors (Basel). 2026 Jan 29;26(3):880. doi: 10.3390/s26030880 (PMC12899450; doi:10.3390/s26030880)
Supplement: Supplementary file 1 [file sensors-26-00880-s001.zip › sensors-4076747-supplementary.pdf]

Supplementary Information

Biocompatible and Flexible Cellulose Film for the Reversible Colourimetric Monitoring of pH and Mg (II)

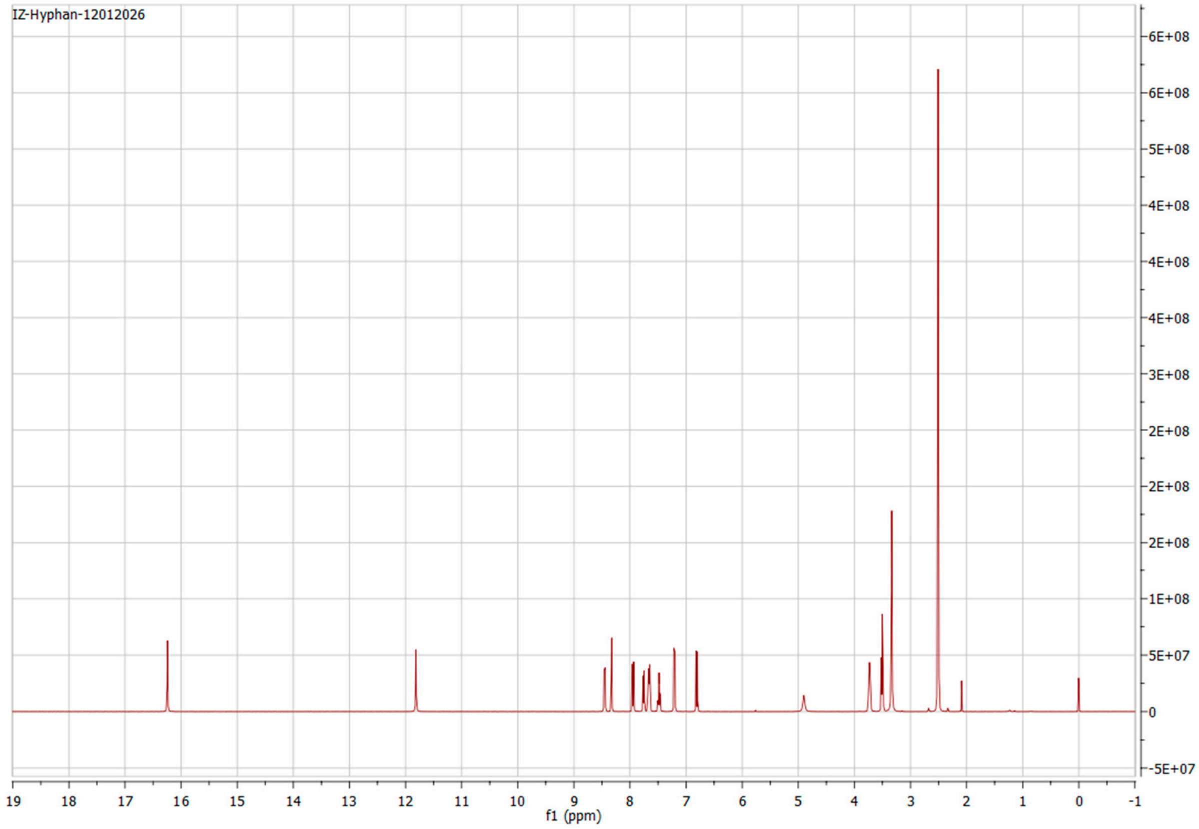

Figure S1. ^{1}H-NMR spectrum of Hyphan I.

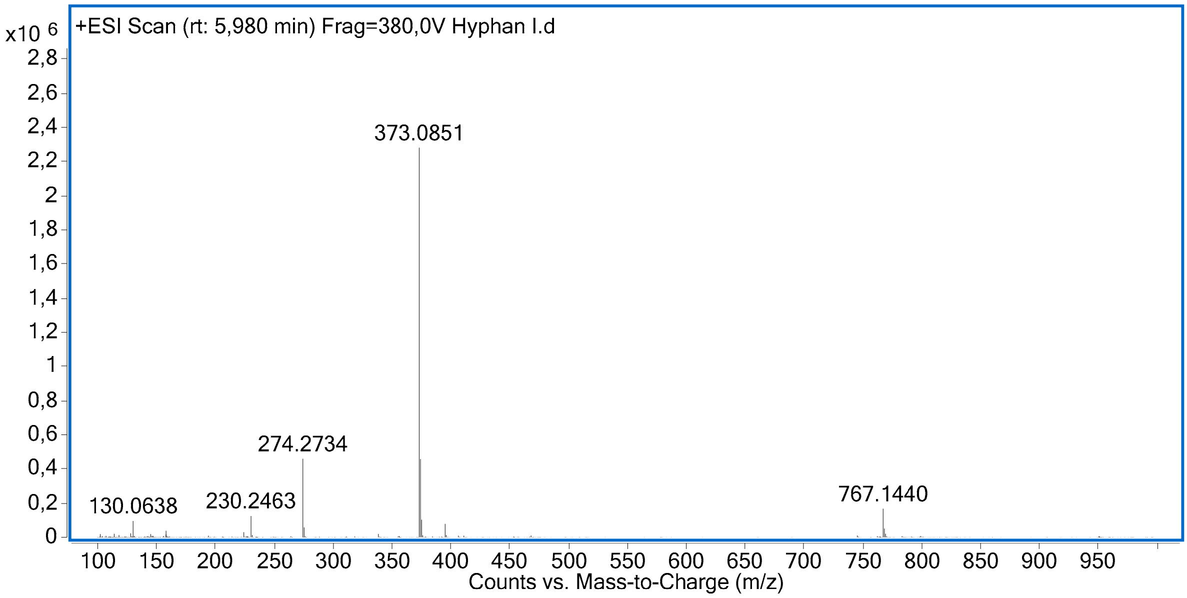

Figure S2. Mass spectrum of Hyphan I.

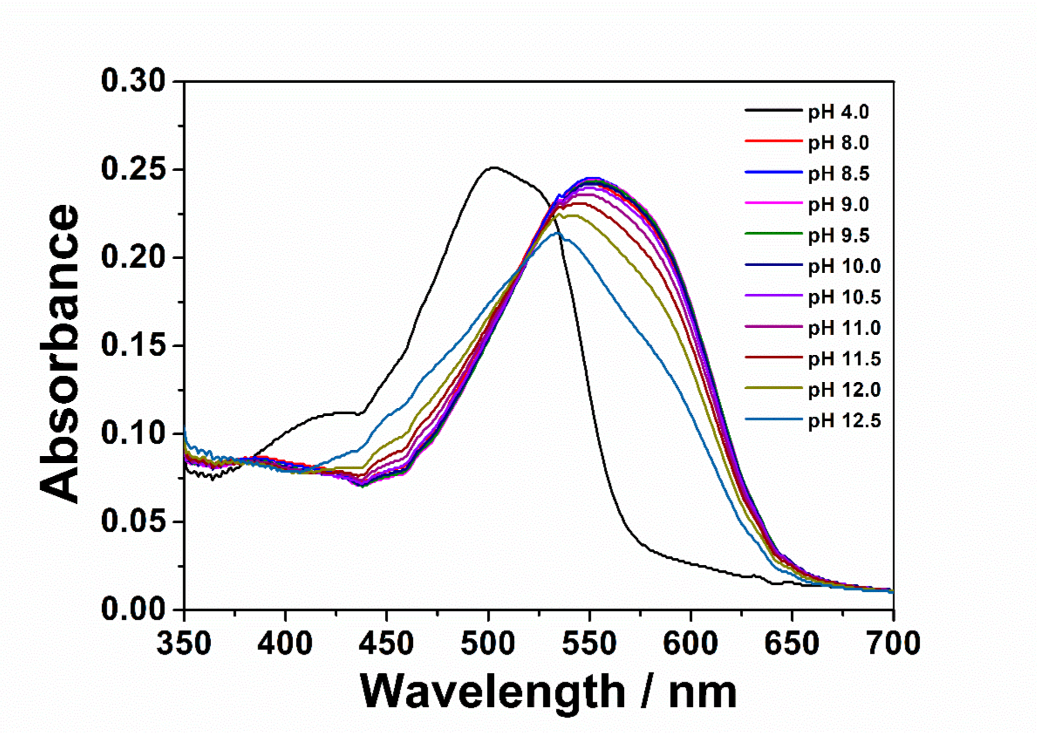

Figure S3. Absorption spectra of Hyphan I corresponding to its various protonated and deprotonated forms.

Section S1. pH response

Table S1. CFH absorbance *vs*. pH, 6 repetitions (R1 – R6)

pH	R1	R2	R3	R4	R5	R6	AVERAGE	STDEV	RSD %
**4.0**	0.036	0.036	0.037	0.038	0.037	0.04	0.037	0.0015	4.03
**4.5**	0.049	0.049	0.05	0.051	0.05	0.051	0.050	0.0009	1.79
**5.0**	0.072	0.073	0.072	0.078	0.074	0.075	0.074	0.0023	3.08
**5.5**	0.11	0.11	0.11	0.111	0.108	0.108	0.110	0.0012	1.12
**6.0**	0.153	0.154	0.154	0.155	0.152	0.152	0.153	0.0012	0.79
**6.5**	0.195	0.198	0.195	0.194	0.191	0.19	0.194	0.0029	1.51
**7.0**	0.227	0.227	0.227	0.223	0.219	0.22	0.224	0.0037	1.66
**7.4**	0.237	0.239	0.239	0.235	0.232	0.231	0.236	0.0034	1.46
**8.0**	0.25	0.251	0.25	0.248	0.241	0.241	0.247	0.0046	1.87
**8.5**	0.254	0.255	0.255	0.25	0.245	0.245	0.251	0.0048	1.90
**9.0**	0.257	0.255	0.257	0.25	0.247	0.243	0.252	0.0058	2.30

Section S2. Mg^{2+} response

Table S2. CFH absorbance *vs*. Mg^{2+} in solution pH 8.0, 6 repetitions (R1 – R6)

$[Mg^{2+}]$ / mM	R1	R2	R3	R4	R5	R6	AVERAGE	STDEV	RSD %
**0**	0.258	0.257	0.256	0.258	0.256	0.258	0.257	0.00090	0.35
**0.625**	0.24	0.239	0.237	0.24	0.244	0.24	0.240	0.00208	0.87
**1.25**	0.227	0.227	0.225	0.229	0.231	0.229	0.228	0.00191	0.84
**1.875**	0.218	0.218	0.217	0.22	0.223	0.22	0.219	0.00197	0.90

**2.50**	0.21	0.211	0.209	0.212	0.214	0.212	0.211	0.00160	0.76
**5.00**	0.195	0.195	0.193	0.198	0.198	0.197	0.196	0.00183	0.93

Section S3. Calculation of LOD and LOQ

For the Boltzmann calibration function, the limit of detection (LOD) and limit of quantification (LOQ) were determined and converted to the corresponding concentrations using the following equations

$$y_{LOD} = y_{blank} \pm 3\sigma_{blank} \qquad \textbf{(1)}$$

$$y_{LOQ} = y_{blank} \pm 10\sigma_{blank} \qquad \textbf{(2)}$$

$$x = x_0 + dx.\, ln\left(\frac{A_1 - y}{y - A_2}\right) \qquad \textbf{(3)}$$

where y_{blank} and σ_{blank} are the absorbance and standard deviation corresponding to blank CFH measurements. The calculated LOD and LOQ values of the CFH for Mg^{2+} ions were 0.089 and 0.318 mM, respectively.

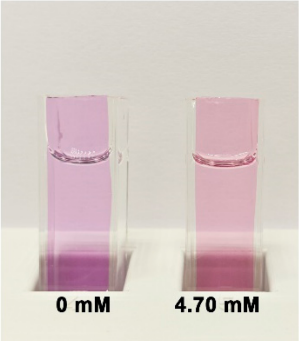

Figure S4. Colourimetric response of CFH to bottled mineral water Mg^{++} Mivela®.
